# Supplementary material for: EGFR is required for FOS‐dependent bone tumor development via RSK2/CREB signaling
Source: EMBO Mol Med. 2018 Oct 25;10(11):e9408. doi: 10.15252/emmm.201809408 (PMC6220323; doi:10.15252/emmm.201809408)
Supplement: Supplementary file 1 — Appendix [file EMMM-10-e9408-s001.pdf]

APPENDIX

EGFR is required for FOS-dependent bone tumor development via RSK2/CREB signaling

Markus Linder[1], Elisabeth Glitzner[1], Sriram Srivatsa[1], Latifa Bakiri[2], Katzuhiko Matsuoka[2], Parastoo Shahrouzi[1], Monika Dumanic[3], Philipp Novoszel[1], Thomas Mohr[1], Oliver Langer[3,4,5], Thomas Wanek[5], Markus Mitterhauser[3,6], Erwin F. Wagner[2] and Maria Sibilia[1,*]

[1]*Institute of Cancer Research, Department of Medicine I, Comprehensive Cancer Center, Medical University of Vienna, Austria*
[2]*Spanish National Cancer Research Center (CNIO), Madrid, Spain*
[3]*Department of Biomedical Imaging and Image-Guided Therapy, Division of Nuclear Medicine, Medical University of Vienna, Vienna, Austria*
[4]*Department of Clinical Pharmacology, Medical University of Vienna, Vienna, Austria*
[5]*Center for Health & Bioresources, AIT Austrian Institute of Technology GmbH, Seibersdorf, Austria*
[6]*LBI Applied Diagnostics, Vienna, Austria*

[*]*Corresponding author:*
Maria Sibilia
Tel: 43-1-40160-57502
Fax: 43-1-40160-957502
Email: Sibilia-Office@meduniwien.ac.at

Table of Contents

- Appendix Figure Legends
- Appendix Figures S1,S2
- Appendix Tables S1,S2

APPENDIX FIGURE LEGENDS

Appendix Figure S1. Myeloid cell-specific EGFR deletion does not affect c-Fos driven osteosarcoma formation.

A. Bone tumor number quantification of 6 month old H2-*c-fos*LTR/Egfr$^{f/f}$ (n=9) and H2-*c-fos*LTR/Egfr$^{f/f}$ *LysM-Cre* mice (n=18).

B. Quantification of tumor size in tibiae. n=8 wt, 19 Egfrf/f LysM-Cre.

C. ALP levels in the serum at 6 months endpoint. n= 9 wt, 18 Egfr$^{f/f}$ *LysM-Cre* mice.

Appendix Figure S2. Amphiregulin over-expression in bone tumors of H2-*c-fos*LTR/*ColAREG* mice leads to elevated EGFR activation but does not affect transgenic c-fos

A. Amphiregulin ELISA (n≥4) in tumor protein lysates and

B. *Areg* qPCR analysis of RNA from tumors of H2-*c-fos*LTR (n=8) and H2-*c-fos*LTR/*ColAREG* mice (n=13).

C. Western Blot analysis of H2-*c-fos*LTR/*ColAREG* tumor lysates.

D. qPCR analysis of transgenic *c-fos* (*Fos*^{tg}) mRNA levels in bone tumor lysates derived from H2-*c-fos*LTR (n=6) or H2-*c-fos*LTR/*ColAREG* mice (n=8).

Appendix Figure S1:

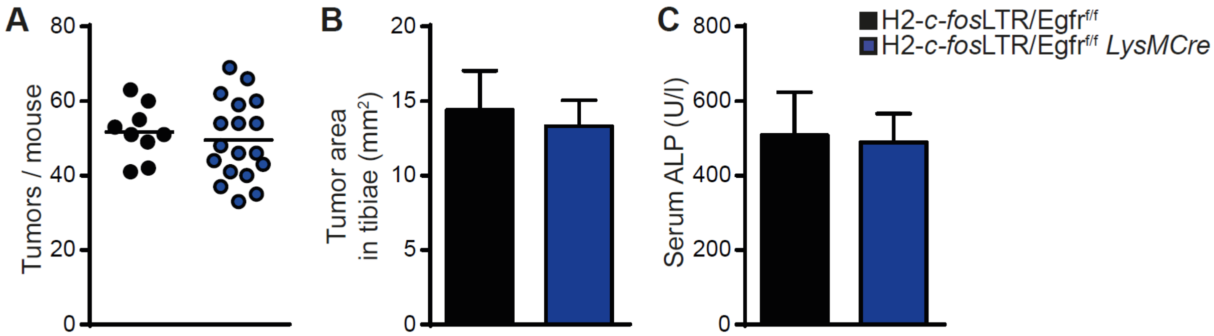

Appendix Figure S2:

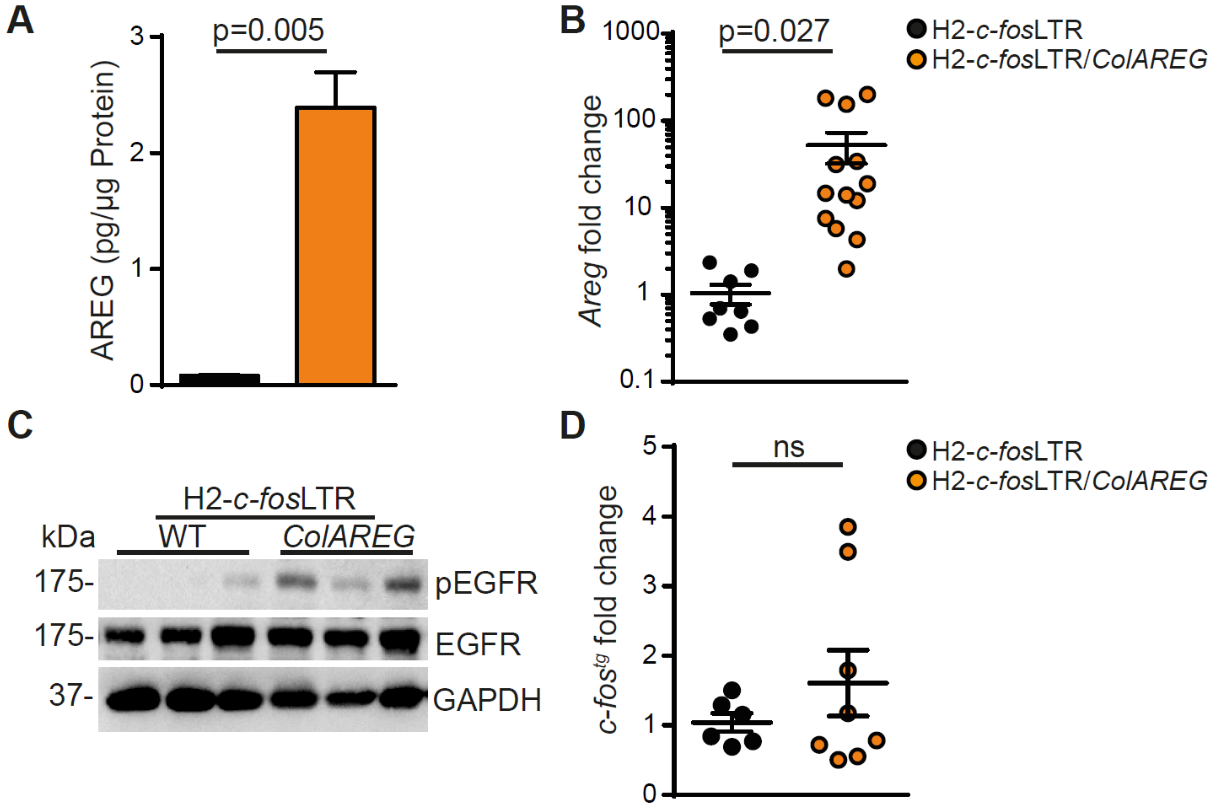

Appendix Table S1:

Antibodies used for Western Blot

DIRECTED AGAINST	COMPANY	PRODUCT NO.	SOURCE	DIUTION
pAKT (T308) (D25E6)	Cell Signaling	13038	Rabbit	1:1000
Caspase 3	Cell Signaling	9662	Rabbit	1:1000
c-Fos (E-8)	Santa Cruz	sc-166940	Mouse	1:200
c-Fos (9F6)	Cell Signaling	2250	Rabbit	1:1000
p-c-Fos (S362)	AAT Bioquest	8A0429	Rabbit	1:1000
cleaved Caspase 3 (Asp175) (5A1E)	Cell Signaling	9664	Rabbit	1:1000
CREB (D76D11)	Cell Signaling	4820	Rabbit	1:1000
pCREB (S133) (87G3)	Cell Signaling	9198	Rabbit	1:1000
CyclinD1 (92G2)	Cell Signaling	2978	Rabbit	1:1000
CyclinD1 (H-295)	Santa Cruz	sc-753	Rabbit	1:500
EGFR	Millipore	06-847	Rabbit	1:500
EGFR (D38B1)	Cell Signaling	4267	Rabbit	1:1000
pEGFR (T1068) (D7A5)	Cell Signaling	3777	Rabbit	1:1000
pERK1/2 (T202/204) (D13.14.E4)	Cell Signaling	4370	Rabbit	1:2000
GAPDH (D16H11)	Cell Signaling	5174	Rabbit	1:5000
HSP90	Cell Signaling	4874	Rabbit	1:1000
PARP (46D11)	Cell Signaling	9532	Rabbit	1:1000
pRSK2 (S227) (D53A11)	Cell Signaling	3556	Rabbit	1:1000
RSK2 (D21B2)	Cell Signaling	5528	Rabbit	1:1000
pS6 (S235/236) (D57.2.2E)	Cell Signaling	4858	Rabbit	1:2000
Tubulin	Sigma	T9026	Mouse	1:500
Vinculin (hVIN-1)	Sigma	V9131	Mouse	1:400

Antibodies used for IHC / IF

DIRECTED AGAINST	COMPANY	PRODUCT NO.	SOURCE	DIUTION
c-Fos	Thermo Fisher	OSC00040W	Rabbit	1:200
c-Fos	Santa Cruz	sc-52	Rabbit	1:500
p-c-Fos (S362)	Bioss	bs-12910R	Rabbit	1:200
cleaved Caspase 3 (Asp175) (5A1E)	Cell Signaling	9664	Rabbit	1:200
pCREB (S133) (87G3)	Cell Signaling	9198	Rabbit	1:800
pEGFR (Y1068)	Cell Signaling	2234	Rabbit	1:400
EGFR (D38B1)	Cell Signaling	4267	Rabbit	1:50
Ki-67	abcam	ab1558	Rabbit	1:1000
PCNA	Cell Signaling	13110	Rabbit	1:5000
pRSK2 (D53A11) (S227)	Cell Signaling	3556	Rabbit	1:100

Appendix Table S2:

Appendix Table S2:

Gene	Forward Primer	Reverse Primer
Amphiregulin (Areg)	AAGAAAACGGGACTGTGCAT	GGCTTGGCAATGATTCAACT
Betacellulin (Btc)	GACGAGCAAACTCCCTCCT	ATCAAGCAGACCACCAGGAT
c-fos	ATGGTGAAGACCGTGTCAGG	GTTGATCTGTCTCCGCTTGGA
human c-fos	CTACCACTCACCCGCAGACT	AGGTCCGTGCAGAGGTCCT
Collagen type 1 alpha 1 (Col1a1)	ACCTGGTCCACAAGGTTTCC	GACCCATTGGACCTGAACCG
Collagen type 1 alpha 2 (Col1a2)	GGTCCAAGAGGAGAACGTGG	TGGGACCTCGGCTTCCAATA
CyclinD1 (Ccnd1)	CGCGTACCCTGACACCAATC	GGAAGACCTCCTCTTCGCAC
Epidermal growth factor (Egf)	TTTCCTTAACGGGACAGGAC	CCCAGAGAACTTCATCATCTATCC
Epidermal growth factor receptor (Egfr)	TTGGAATCAATTTTACACCGAAT	GTTCCCACACAGTGACACCA
human Epidermal growth factor receptor (Egfr)	GCCTTGACTGAGGACAGCA	TTTGGGAACGGACTGGTTA
Epiregulin (Ereg)	CACCGAGAAAGAAGGATGGA	TCAGGTTGTGCTGATAACTG
Heparin-binding EGF-like growth factor (Hb-egf)	CCAGTGGAGAATCCCCTATAC	GCCAAGACTGTAGTGTGGTCA
Osteocalcin (Ocn)	AGACTCCGGCGCTACCTT	CTCGTCACAAGCAGGGTTAAG
Osteonectin (On)	TCTCAAAGTCTCGGGCCAAC	ATGCAAATACATCGCCCCCT
Osteopontin (Opn)	CTGGCTGAATTCTGAGGGACT	TTCTGTGGCGCAAGGAGATT
Osterix (Osx)	TGCCTGACTCCTTGGGACC	TAGTGAGCTTCTTCCTCAAGCA
Runt-related transcription factor 2 (Runx2)	GCCGGGAATGATGAGAACTA	GGACCGTCCACTGTCACTTT
TATA-binding protein (Tbp)	GGGGAGCTGTGATGTGAAGT	CCAGGAAATAATTCTGGCTCAT
Transforming growth factor alpha (Tgfa)	TCTGGGTACGTGGGTGTTC	ACAGGTGATAATGAGGACAGCC
transgenic c-fos (c-fos^{tg})	TGTGTTCCTGGCAATAGCGTGT	GGCAATTCCGCCCATAGTGA
