# Supplementary material for: EGFR is required for FOS‐dependent bone tumor development via RSK2/CREB signaling
Source: EMBO Mol Med. 2018 Oct 25;10(11):e9408. doi: 10.15252/emmm.201809408 (PMC6220323; doi:10.15252/emmm.201809408)
Supplement: Supplementary file 2 — Expanded View Figures PDF [file EMMM-10-e9408-s002.pdf]

Expanded View Figures

Figure EV1. H2-*c-fos*LTR mice show elevated mRNA expression of *Egfr* and *c-fos* and upregulation of the EGFR ligands *Hb-EGF* and *TGFa*.

A *Egfr* mRNA expression in normal wildtype (wt) bones and in normal bones or OSs of H2-*c-fos*LTR mice (n = 4).
B *c-fos* mRNA expression in normal wt bones and in normal bones or OSs of H2-*c-fos*LTR mice (n = 4).
C Erlotinib treatment scheme: 2-month-old H2-*c-fos*LTR/Egfr^{wt} mice were treated with vehicle or erlotinib (25 mg/kg, 5x/week for 3 x 4 weeks with 2 x 4 week non-treatment interval in-between). X-ray analysis and blood sampling for tumor number/size quantification and serum ALP measurement were performed once a month.
D pEGFR IHC analysis of OSs from 7-month-old H2-*c-fos*LTR/Egfr^{wt} mice after vehicle or erlotinib treatment. Scale bars: 500 μm (lower magnification), 100 μm (higher magnification).
E EGFR ligand mRNA expression in OS from H2-*c-fos*LTR mice (n = 4), normalized to *Egf*.
F EGFR ligand mRNA expression in human OS (n = 42, data analyzed from publicly available dataset E-GEOD-39058).
G *Hb-EGF* mRNA expression in normal wildtype (wt) bones and in normal bones or OSs of H2-*c-fos*LTR mice (n = 4).
H *TGFa* mRNA expression in normal wildtype (wt) bones and in normal bones or OSs of H2-*c-fos*LTR mice (n = 4).

Data information: Data are shown as mean ± SEM. *P*-values were calculated by one-way ANOVA followed by Bonferroni multiple comparison test.

 © 2018 The Authors

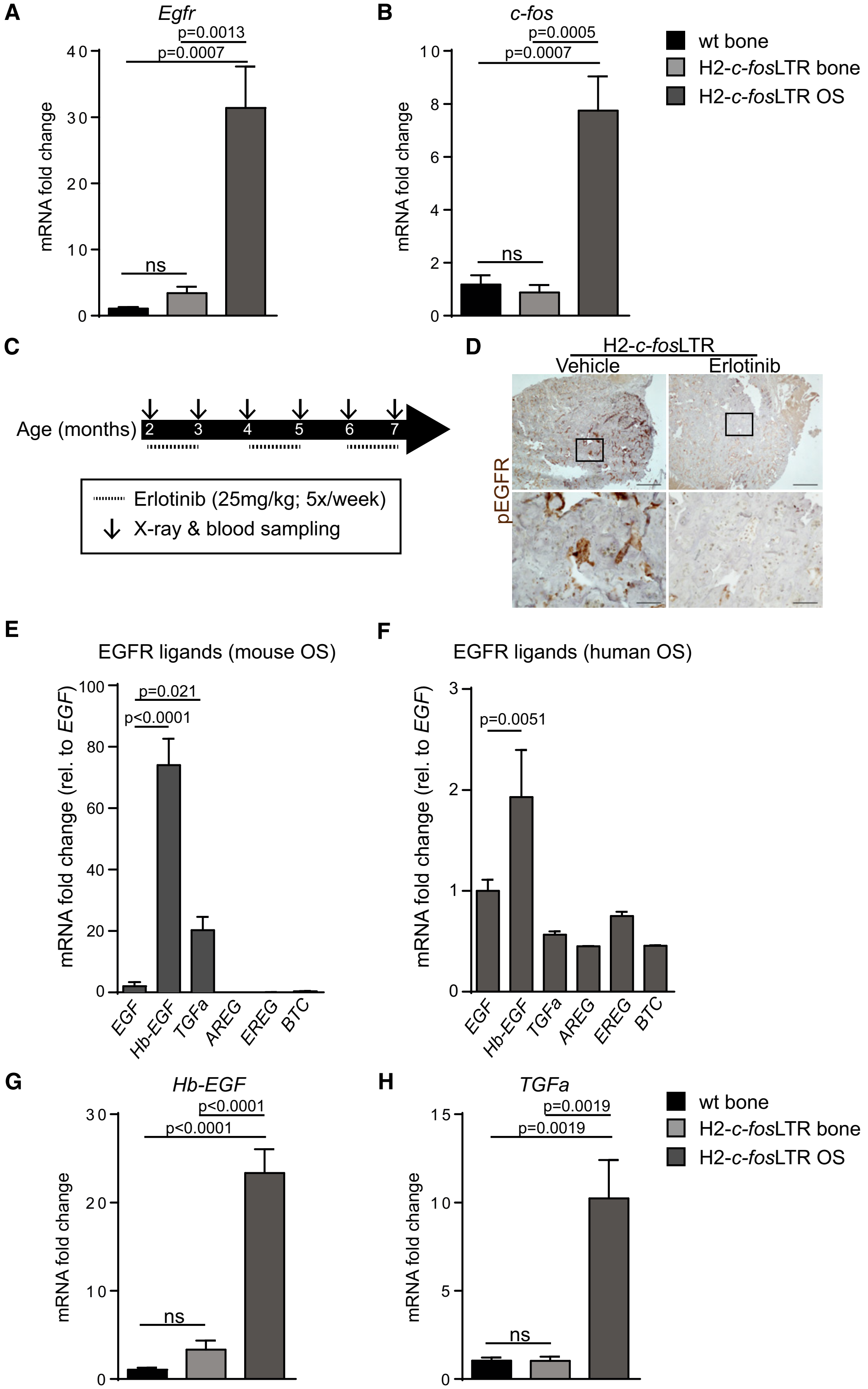
A
Egfr
p=0.0013
p=0.0007
mRNA fold change
ns
B
c-fos
p=0.0005
p=0.0007
mRNA fold change
ns
wt bone
H2-c-fosLTR bone
H2-c-fosLTR OS
C
Age (months)
Erlotinib (25mg/kg; 5x/week)
X-ray & blood sampling
D
H2-c-fosLTR
Vehicle
Erlotinib
pEGFR
E
EGFR ligands (mouse OS)
p=0.021
p<0.0001
mRNA fold change (rel. to EGF)
EGF
Hb-EGF
TGFa
AREG
EREG
BTC
F
EGFR ligands (human OS)
p=0.0051
mRNA fold change (rel. to EGF)
G
Hb-EGF
p<0.0001
p<0.0001
mRNA fold change
ns
H
TGFa
p=0.0019
p=0.0019
mRNA fold change
ns
wt bone
H2-c-fosLTR bone
H2-c-fosLTR OS

Figure EV1.

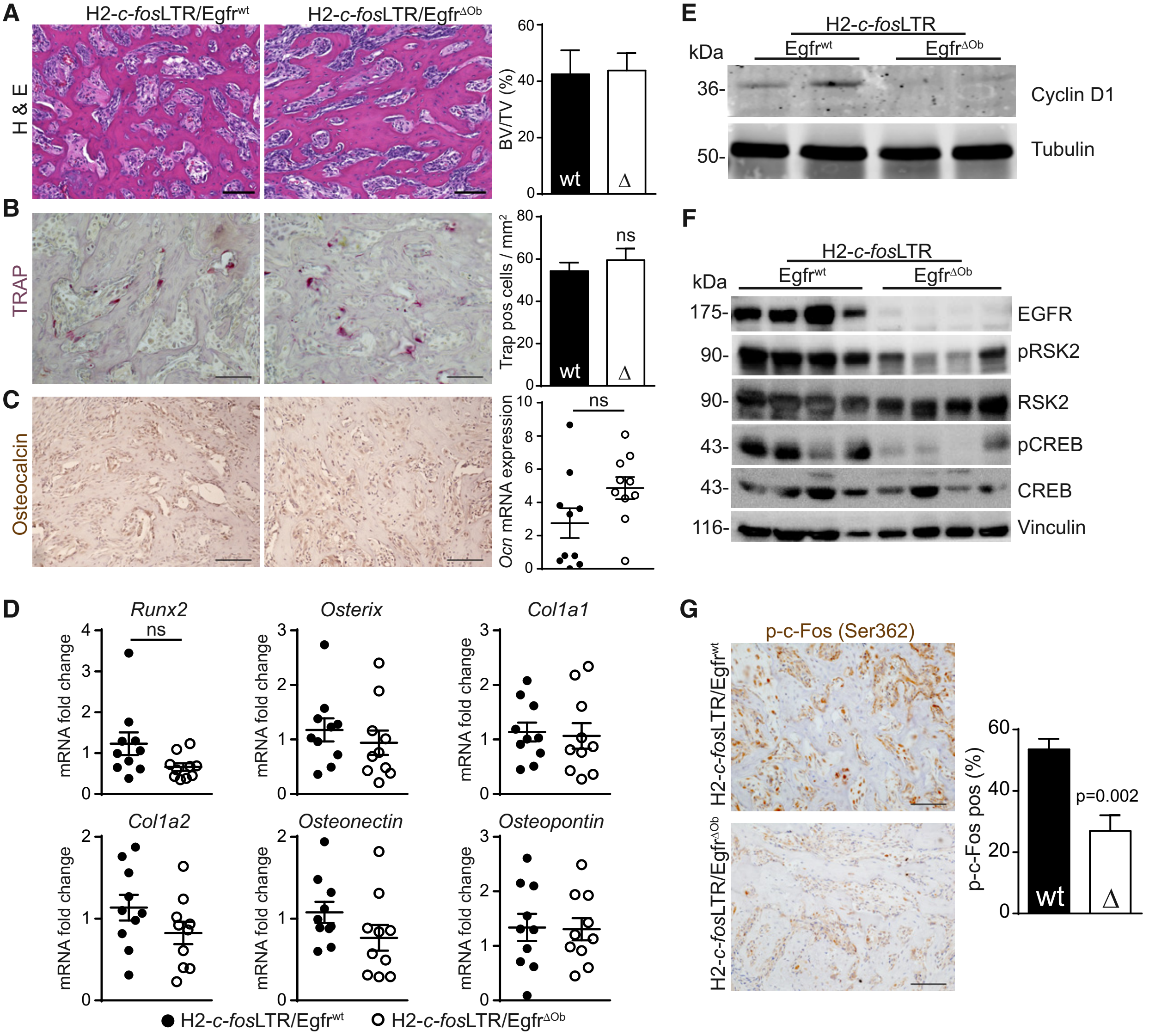

Figure EV2. EGFR deletion does not affect OS morphohistology but leads to reduced proliferation and RSK2-dependent phosphorylation of c-Fos at Ser362.

A H&E staining of H2-*c-fos*LTR/Egfr^{wt} and H2-*c-fos*LTR/Egfr$^{\Delta Ob}$ bone tumors and quantification of bone volume to total volume (BV/TV) in OS. n = 6, scale bars: 100 μm.

B TRAP staining and quantification. n = 5 wt, 4 ΔOb, scale bars: 100 μm.

C Osteocalcin IHC staining and mRNA analysis of bone tumors of H2-*c-fos*LTR/Egfr^{wt} and H2-*c-fos*LTR/Egfr$^{\Delta Ob}$ mice (n = 10). Scale bars: 100 μm.

D mRNA expression analysis of whole OS RNA isolated from 6-month-old H2-*c-fos*LTR/Egfr^{wt} and H2-*c-fos*LTR/Egfr$^{\Delta Ob}$ mice (n = 10).

E Cyclin D1 Western blot analysis of whole OS protein lysates.

F Western blot analysis of whole OS protein lysates isolated from 6- to 7-month-old H2-*c-fos*LTR/Egfr^{wt} and H2-*c-fos*LTR/Egfr$^{\Delta Ob}$ mice.

G p-c-Fos (Ser362) IHC staining and quantification shown as % positive cells in OS from H2-*c-fos*LTR/Egfr^{wt} and H2-*c-fos*LTR/Egfr$^{\Delta Ob}$ mice. n = 6, scale bars: 100 μm.

Data information: Data are shown as mean ± SEM. *P*-values were calculated by unpaired, two-tailed *t*-test.
Source data are available online for this figure.

© 2018 The Authors

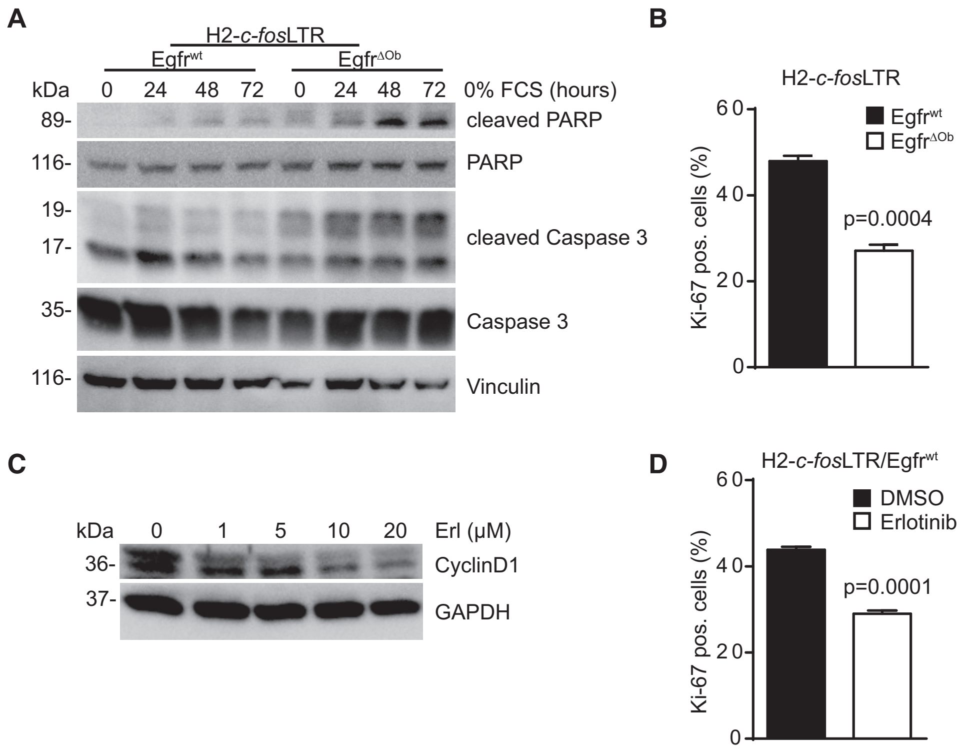

Figure EV3. EGFR is essential for proliferation and survival of *c-fos* transgenic OS cells.

A Western blot analysis of H2-*c-fos*LTR/Egfr^{wt} and H2-*c-fos*LTR/Egfr$^{\Delta Ob}$ OS cells starved for indicated time-points.
B Quantification of Ki-67-positive primary H2-*c-fos*LTR/Egfr^{wt} and H2-*c-fos*LTR/Egfr$^{\Delta Ob}$ OS cells (n = 3).
C Western blot analysis of H2-*c-fos*LTR/Egfr^{wt} OS cells treated for 24 h with erlotinib as indicated.
D Quantification of Ki-67-positive H2-*c-fos*LTR/Egfr^{wt} OS cells after 24-h treatment with erlotinib (10 μM; n = 3).

Data information: Data are shown as mean ± SEM. *P*-values were calculated by unpaired, two-tailed *t*-test.
Source data are available online for this figure.

© 2018 The Authors

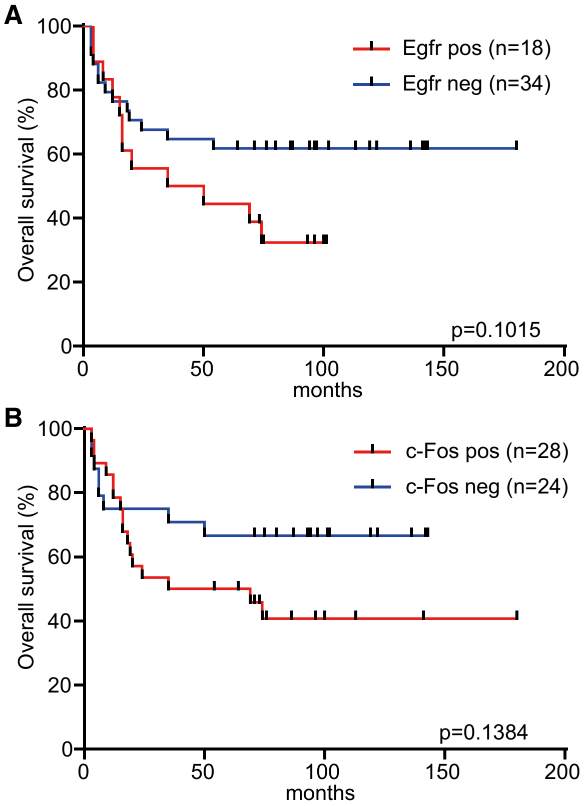

C

EGFR ligands

correation with *FOS* expression in human OS		
Gene	Pearson correlation coefficient	*p*-value
AREG	0.031	0.84
BTC	-0.086	0.59
EGF	-0.18	0.25
EREG	**-0.39**	**0.011**
HBEGF	-0.11	0.47
TGFA	**0.45**	**0.0029**

Pearson Correlation: 1.0, 0.5, 0, -0.5, -1.0

Figure EV4. EGFR or c-Fos expression does not significantly correlate with patient survival.

A Kaplan–Meier survival curve correlating EGFR protein levels in the bone tumors with patient survival. $n = 52$.

B Kaplan–Meier survival curve correlating c-Fos protein levels in the bone tumors with patient survival. $n = 52$.

C Gene expression correlation analysis of EGFR ligands with *FOS* in human OS (data analyzed from publicly available dataset E-GEOD-39058).

Data information: *P*-values were calculated by log-rank (Mantel–Cox) test comparing the two Kaplan–Meier curves (A and B) or unpaired, two-tailed *t*-test (C).

Figure EV5. c-Fos is essential for 143b cell growth, cell viability, and proliferation.

A Relative *Egfr* and *c-fos* mRNA expression levels of different human OS cell lines, cultured under standard conditions.

B Western blot analysis of starved LM7 cells, pre-treated with DMSO (1:1,000) or afatinib (5 μM) for 30 min and stimulated with EGF (50 ng/ml) as indicated.

C, D *c-fos* mRNA (C) and c-Fos protein expression analysis (D) of 143b cells harboring doxycycline-inducible c-fos (sh_FOS) or control (sh_Ctrl) shRNA knock-down, cultured with (+) or without (−) doxycycline (0.5 μg/ml).

E, F Cumulative cell growth (E) and MTT assay (F) of 143b sh_Crtl or 143b sh_FOS cells, cultured with (+) or without (−) doxycycline (0.5 μg/ml) ($n = 3$).

G Quantification of cleaved caspase-3-positive 143b sh_Ctrl or 143b sh_FOS cells, cultured for 48 h with (+) or without (−) doxycycline (0.5 μg/ml) ($n = 4/5$).

H Quantification of Ki-67-positive 143b sh_Ctrl or 143b sh_FOS cells, cultured for 48 h with (+) or without (−) doxycycline (0.5 μg/ml) ($n = 6$).

I, J IHC staining showing EGFR (I) and c-Fos (J) protein expression in xenografts derived from either 143b or LM7 tumor cells after intratibial injection. Scale bars: 50 μm.

K pEGFR IHC staining of 143b and LM7 orthotopic xenograft after vehicle or erlotinib treatment. Scale bars: 50 μm.

L IHC analysis of Ki67 in 143b-derived tumors ($n = 4$). Scale bars: 50 μm.

M Western blot analysis showing cyclin D1 protein levels in 143b tumor lysates after vehicle or erlotinib treatment.

Data information: Data are shown as mean ± SEM. *P*-values were calculated by unpaired, two-tailed *t*-test.
Source data are available online for this figure.

© 2018 The Authors

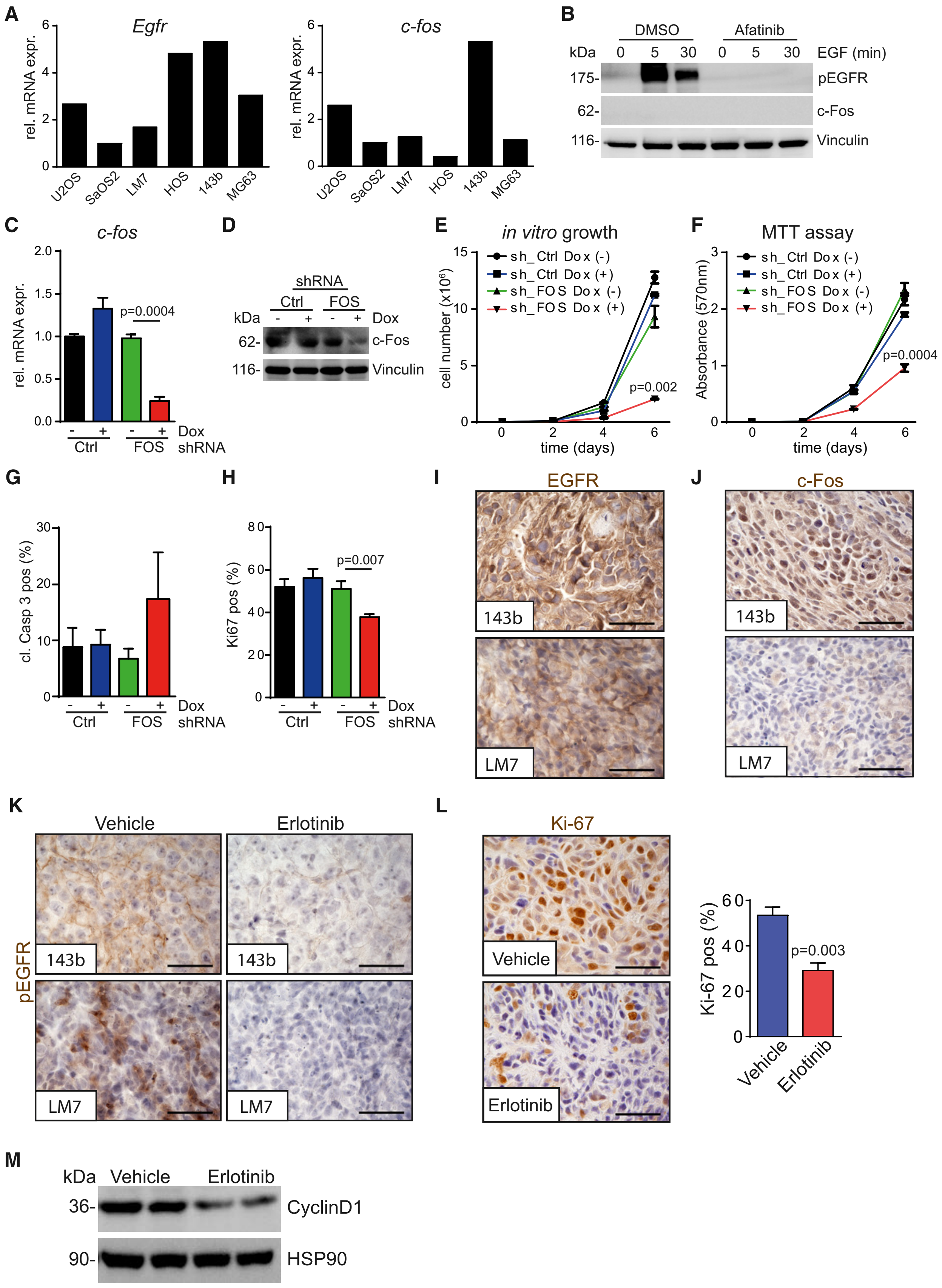
A
Egfr
rel. mRNA expr.
U2OS
SaOS2
LM7
HOS
143b
MG63
c-fos
B
DMSO
Afatinib
kDa
EGF (min)
175-
pEGFR
62-
c-Fos
116-
Vinculin
C
c-fos
rel. mRNA expr.
p=0.0004
Dox
Ctrl
FOS
shRNA
D
shRNA
Ctrl
FOS
kDa
Dox
62-
c-Fos
116-
Vinculin
E
in vitro growth
cell number (x10^6)
sh_Ctrl Dox (-)
sh_Ctrl Dox (+)
sh_FOS Dox (-)
sh_FOS Dox (+)
p=0.002
time (days)
F
MTT assay
Absorbance (570nm)
p=0.0004
time (days)
G
cl. Casp 3 pos (%)
Dox
Ctrl
FOS
shRNA
H
Ki67 pos (%)
p=0.007
Dox
Ctrl
FOS
shRNA
I
EGFR
143b
LM7
J
c-Fos
143b
LM7
K
Vehicle
Erlotinib
pEGFR
143b
LM7
L
Ki-67
Vehicle
Erlotinib
Ki-67 pos (%)
p=0.003
Vehicle
Erlotinib
M
kDa
Vehicle
Erlotinib
36-
CyclinD1
90-
HSP90

Figure EV5.
