# Supplementary material for: EGFR is required for FOS‐dependent bone tumor development via RSK2/CREB signaling
Source: EMBO Mol Med. 2018 Oct 25;10(11):e9408. doi: 10.15252/emmm.201809408 (PMC6220323; doi:10.15252/emmm.201809408)

Unedited blots for Figure EV2E

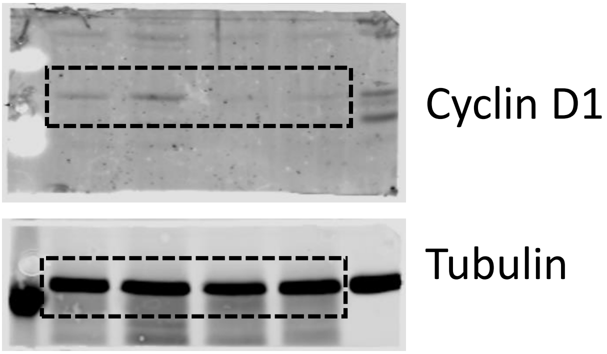

Unedited blots for Figure EV2F

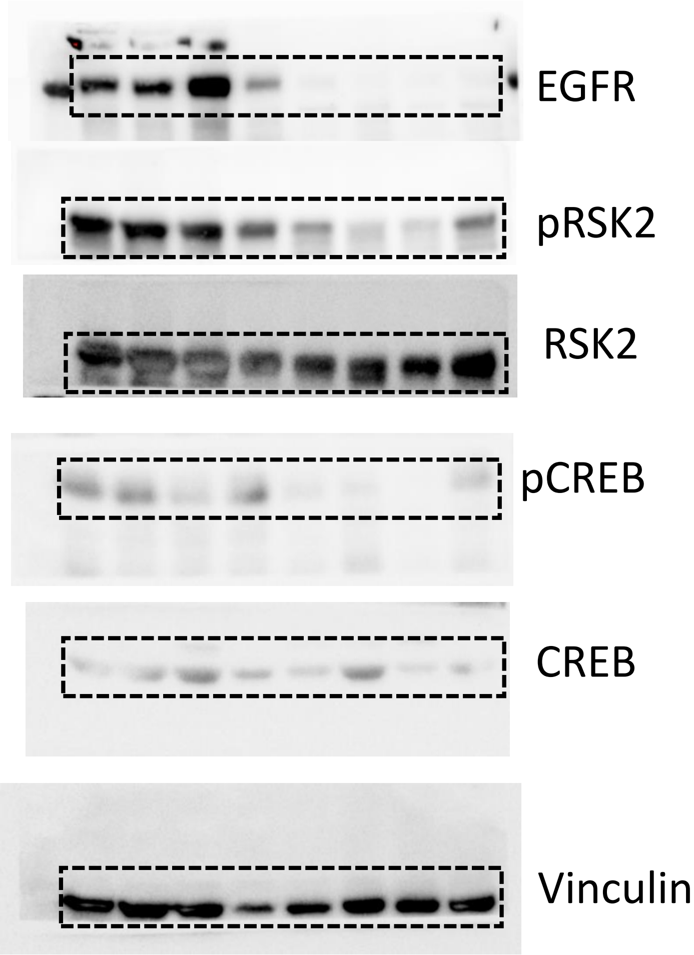

Unedited blots for Figure EV3A

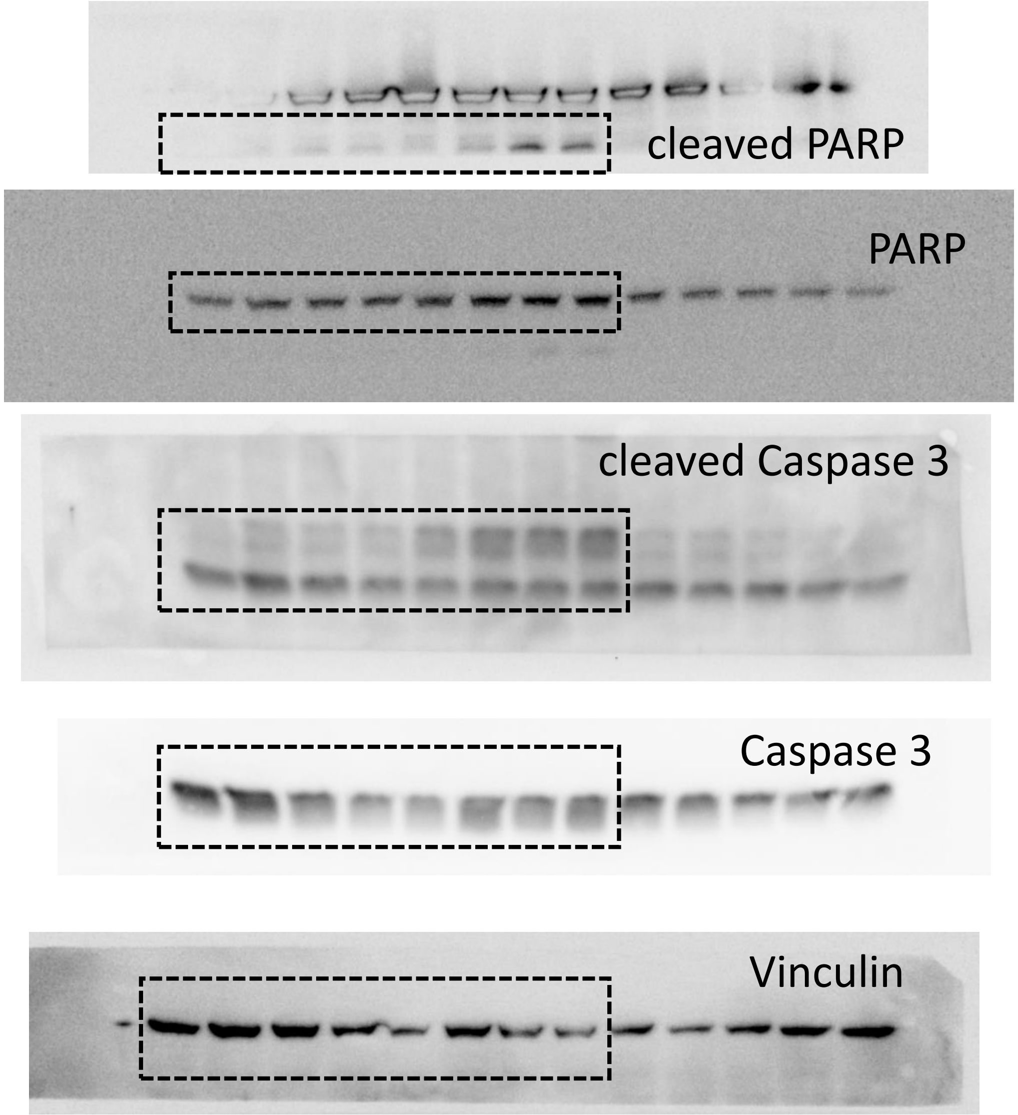

Unedited blots for Figure EV3C

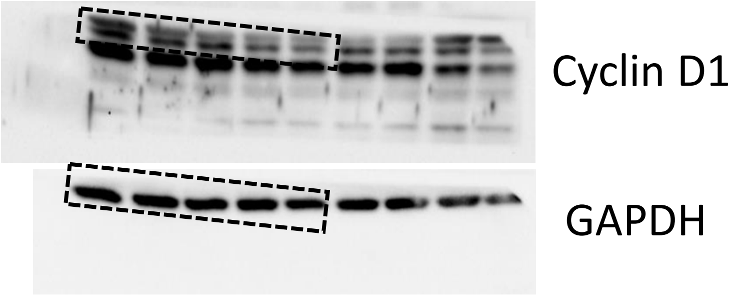

Unedited blots for Figure EV5B

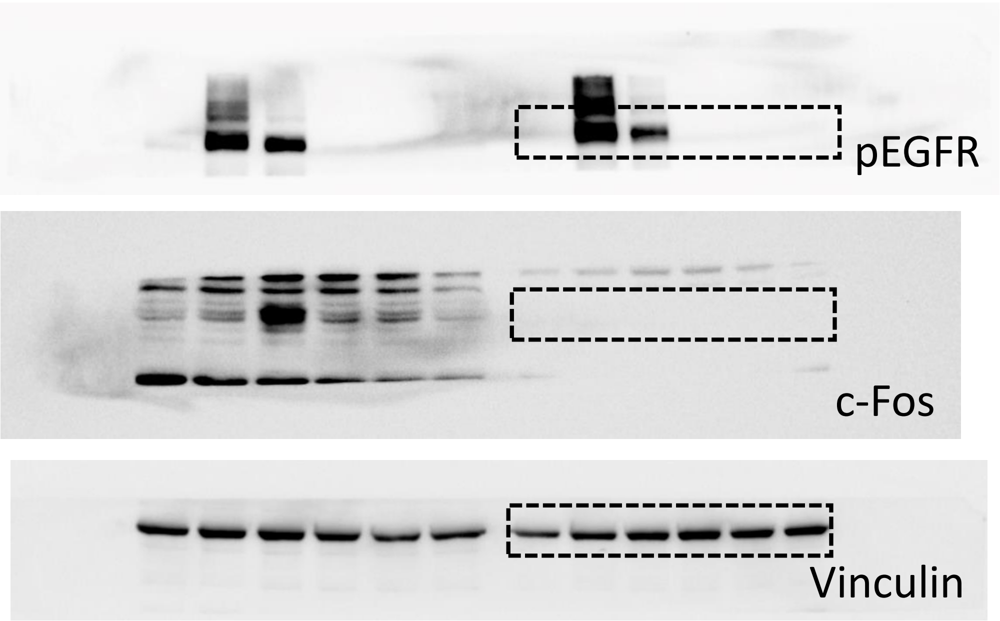

Unedited blots for Figure EV5D

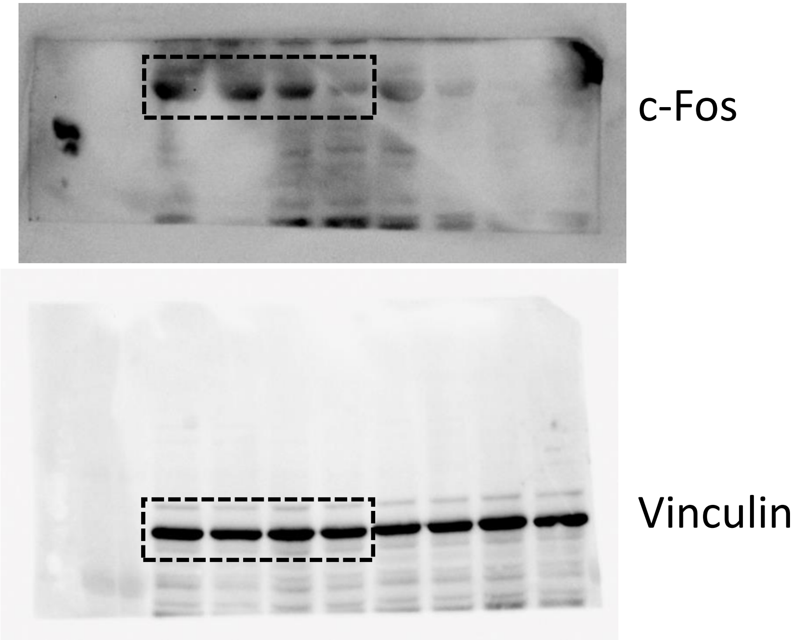

Unedited blots for Figure EV5M

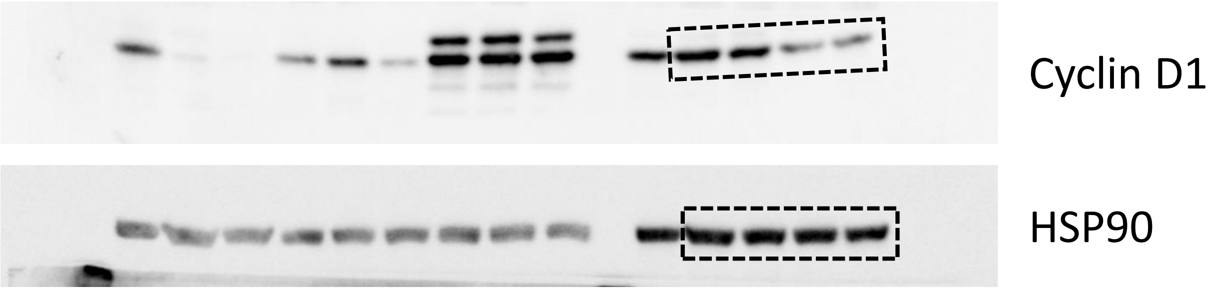

Unedited blots for Appendix Figure S2C

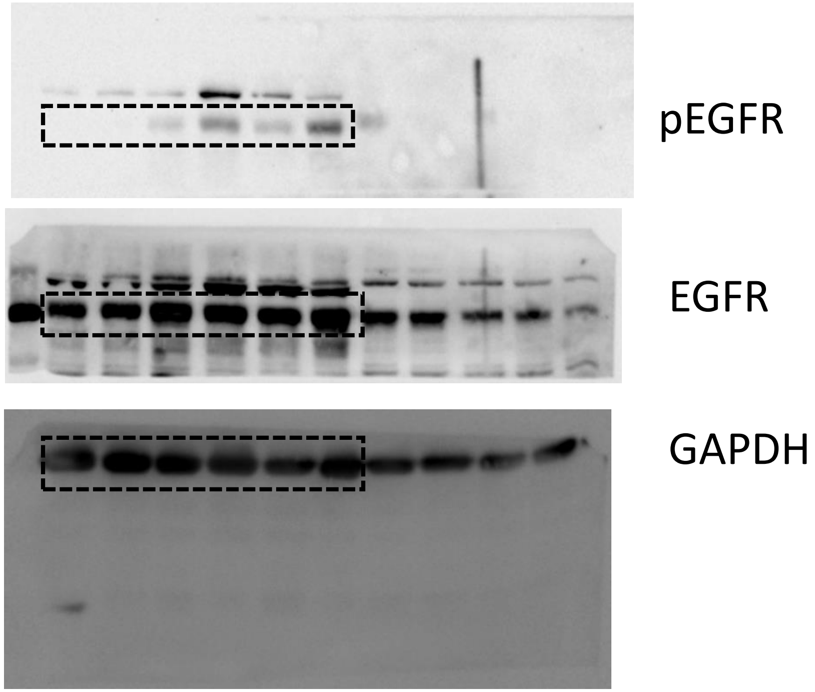

Supplement: Supplementary file 4 — Source Data for Expanded View and Appendix [file EMMM-10-e9408-s007.zip › SourceDataForExpandedViewAndAppendix.pdf]
