# Supplementary material for: EGFR is required for FOS‐dependent bone tumor development via RSK2/CREB signaling
Source: EMBO Mol Med. 2018 Oct 25;10(11):e9408. doi: 10.15252/emmm.201809408 (PMC6220323; doi:10.15252/emmm.201809408)

Unedited blots for Figure 4A

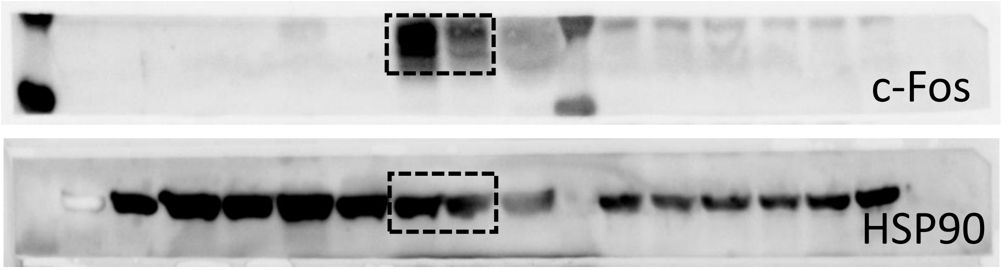

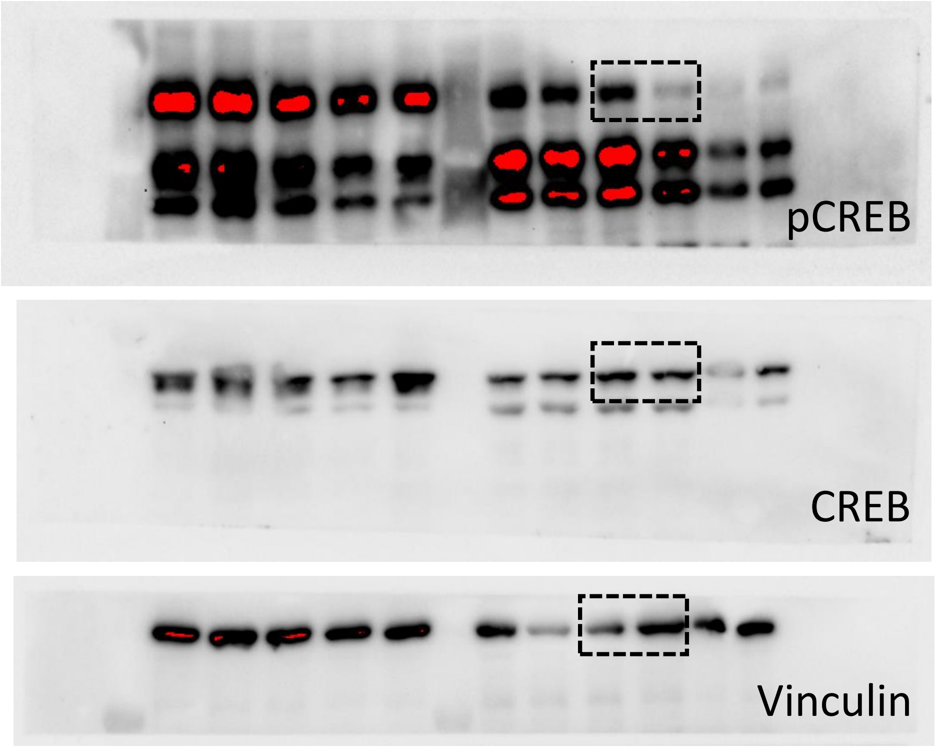

Unedited blots for Figure 4C

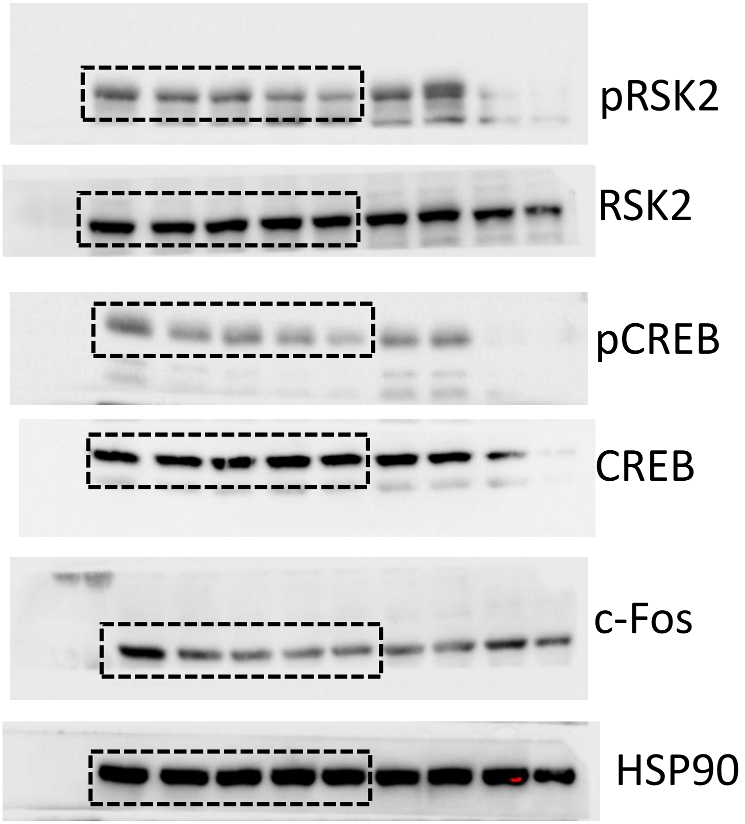

Unedited blots for Figure 4E

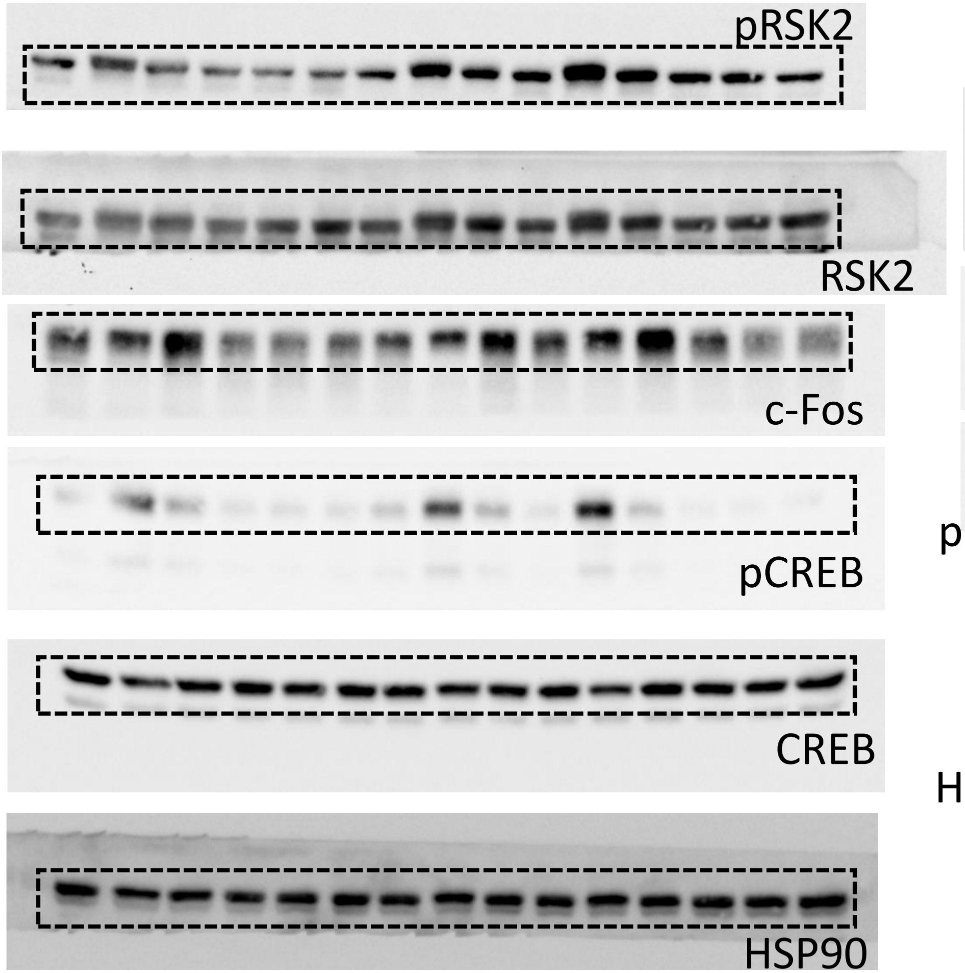

Unedited blots for Figure 4F

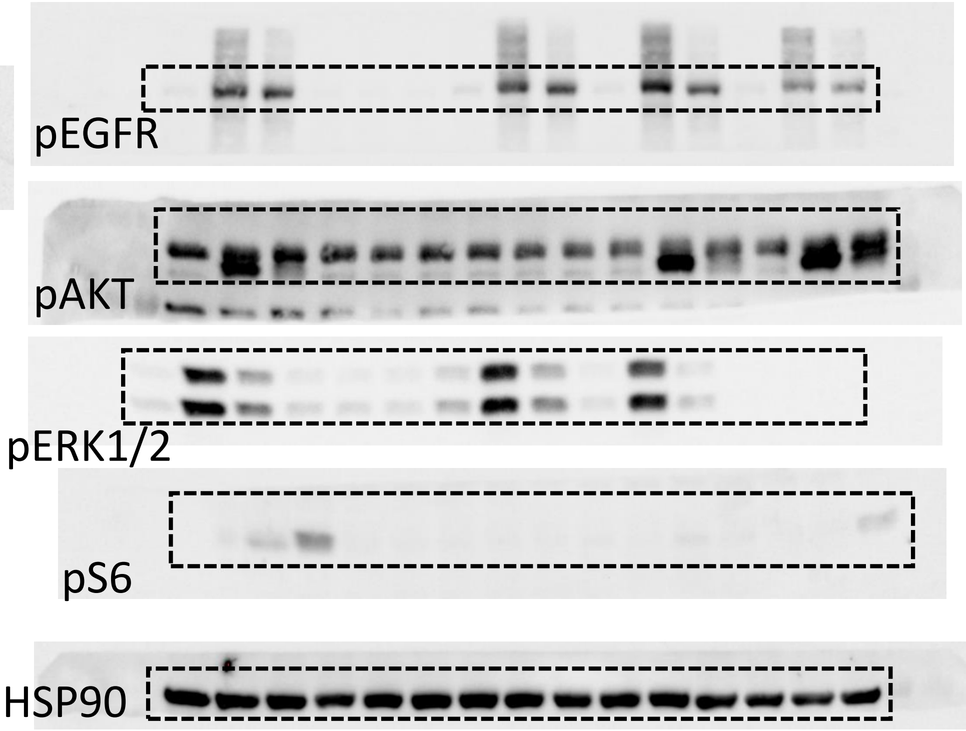

Unedited blots for Figure 4G

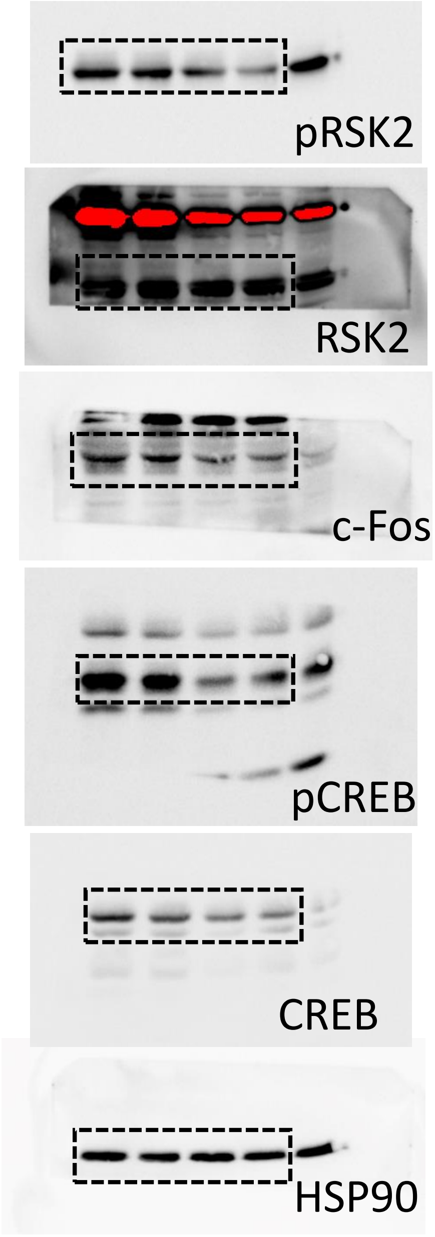

Unedited blots for Figure 4I

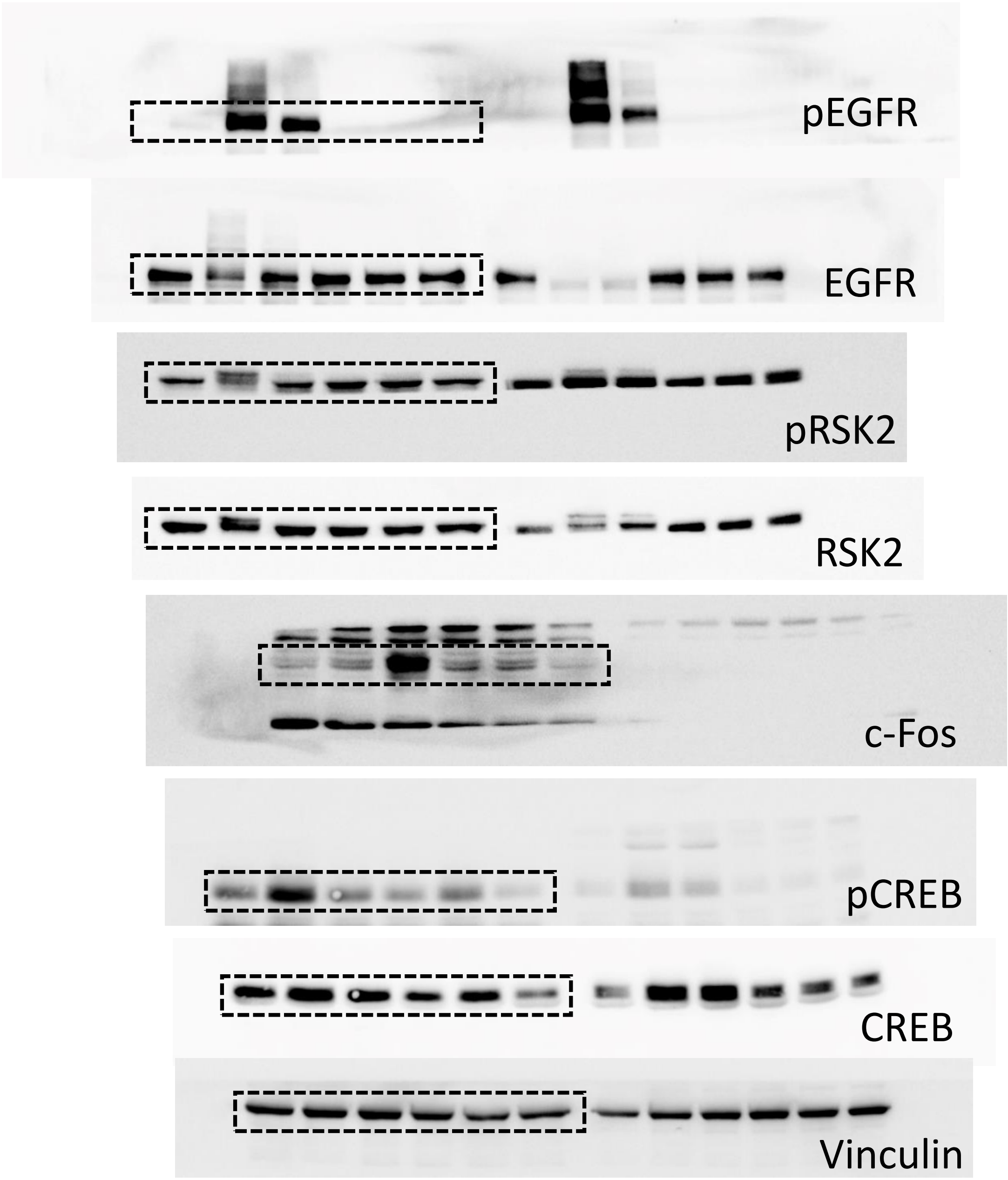

Supplement: Supplementary file 6 — Source Data for Figure 4 [file EMMM-10-e9408-s004.pdf]
