# Supplementary figures and images for: EGFR is required for FOS‐dependent bone tumor development via RSK2/CREB signaling
Source: EMBO Mol Med. 2018 Oct 25;10(11):e9408. doi: 10.15252/emmm.201809408 (PMC6220323; doi:10.15252/emmm.201809408)

Unedited blots for Figure 5F

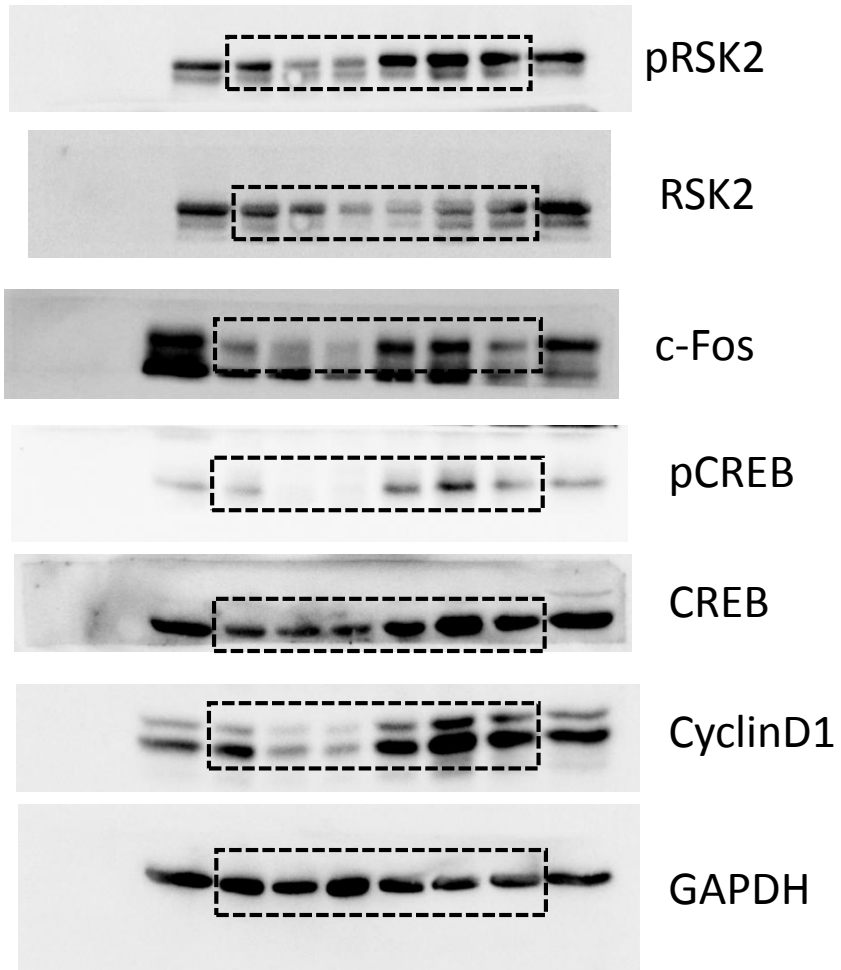

Unedited blots for Figure 5G

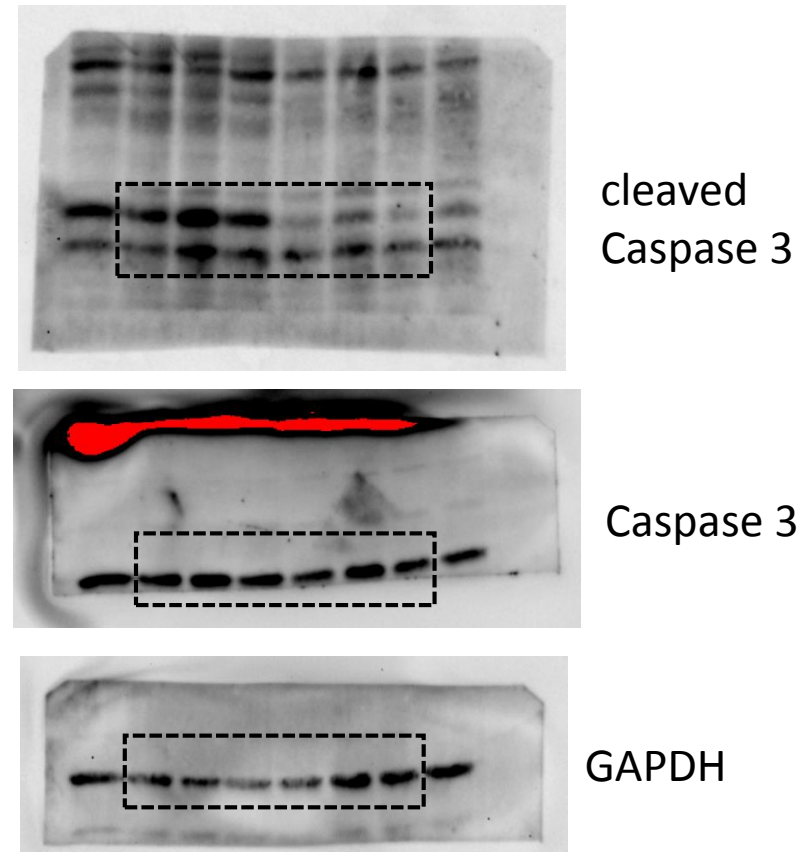

Supplement: Supplementary file 7 — Source Data for Figure 5 [file EMMM-10-e9408-s005.pdf]

# Unedited blots for Figure 7A

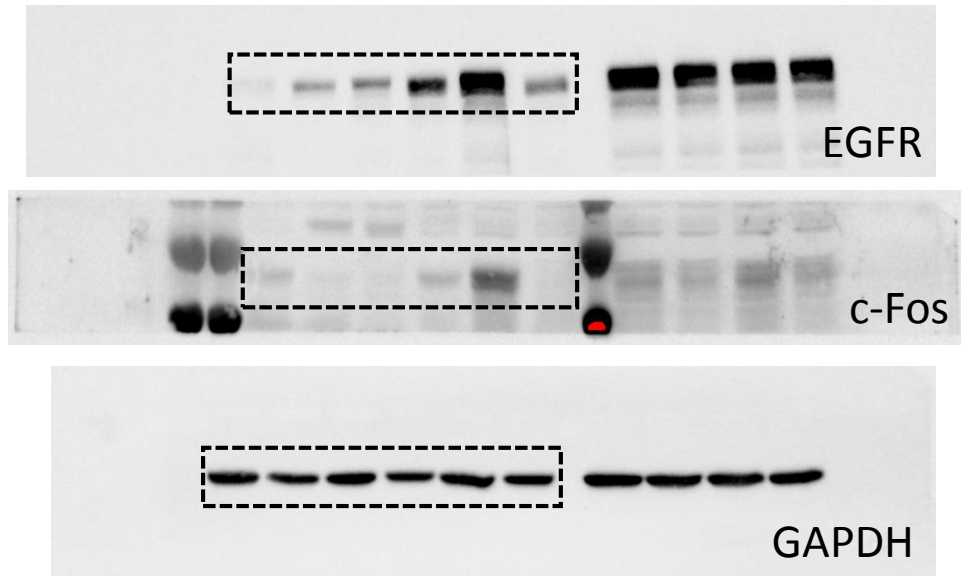

# Unedited blots for Figure 7L

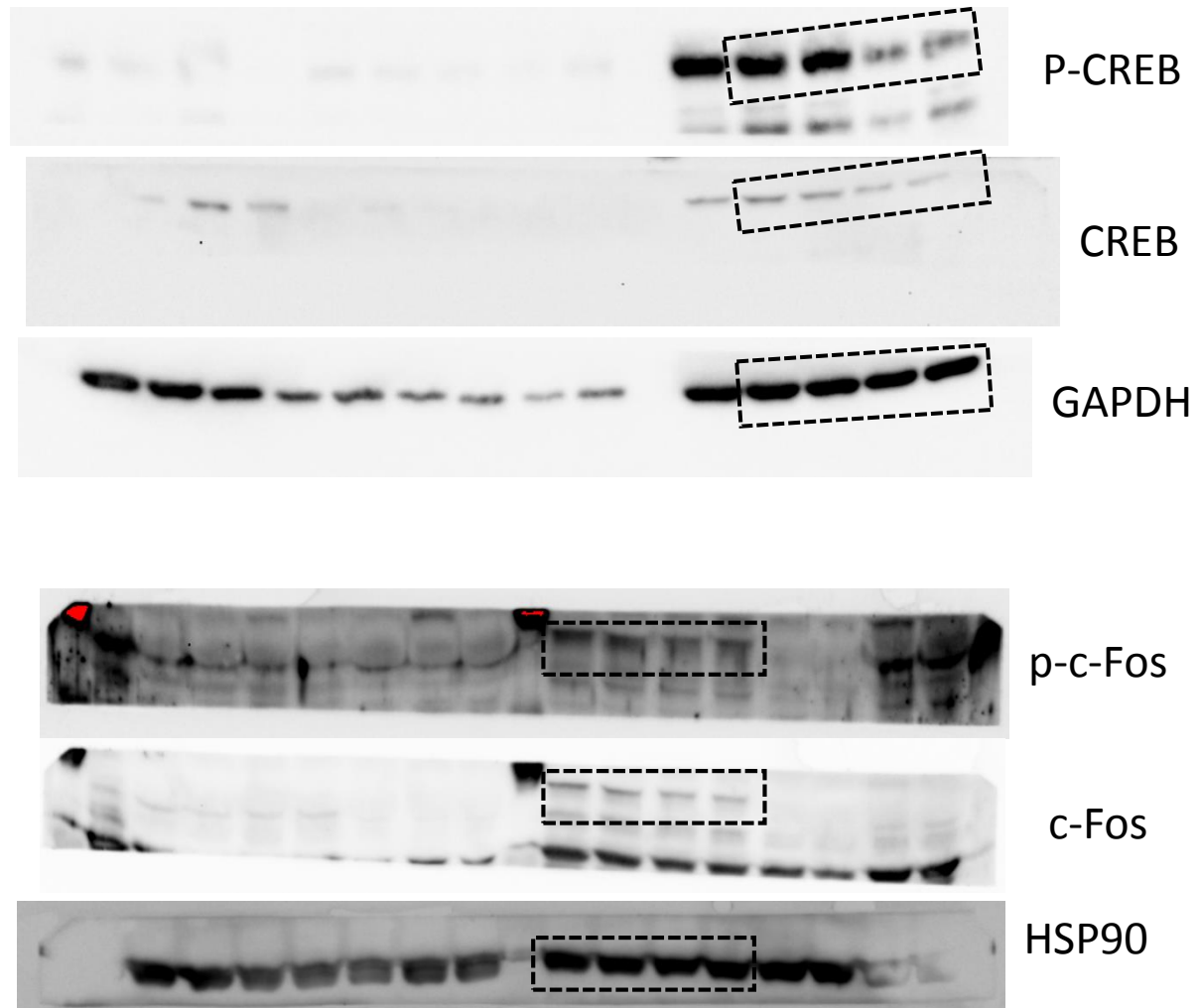

Supplement: Supplementary file 8 — Source Data for Figure 7 [file EMMM-10-e9408-s006.pdf]
